# Epigenetic Regulation of Cell-Fate Changes That Determine Adult Liver Regeneration After Injury

**DOI:** 10.3389/fcell.2021.643055

**Published:** 2021-03-01

**Authors:** Luigi Aloia

**Affiliations:** MRC Laboratory for Molecular Cell Biology, University College London, London, United Kingdom

**Keywords:** plasticity, cell-fate change, epigenetics, liver cancer, chronic liver disease, liver regeneration, liver progenitor, metabolism

## Abstract

The adult liver has excellent regenerative potential following injury. In contrast to other organs of the body that have high cellular turnover during homeostasis (e.g., intestine, stomach, and skin), the adult liver is a slowly self-renewing organ and does not contain a defined stem-cell compartment that maintains homeostasis. However, tissue damage induces significant proliferation across the liver and can trigger cell-fate changes, such as trans-differentiation and de-differentiation into liver progenitors, which contribute to efficient tissue regeneration and restoration of liver functions. Epigenetic mechanisms have been shown to regulate cell-fate decisions in both embryonic and adult tissues in response to environmental cues. Underlying their relevance in liver biology, expression levels and epigenetic activity of chromatin modifiers are often altered in chronic liver disease and liver cancer. In this review, I examine the role of several chromatin modifiers in the regulation of cell-fate changes that determine efficient adult liver epithelial regeneration in response to tissue injury in mouse models. Specifically, I focus on epigenetic mechanisms such as chromatin remodelling, DNA methylation and hydroxymethylation, and histone methylation and deacetylation. Finally, I address how altered epigenetic mechanisms and the interplay between epigenetics and metabolism may contribute to the initiation and progression of liver disease and cancer.

## Introduction

The adult liver has extraordinary regenerative potential. This knowledge dates back to the myth of Prometheus, who was condemned to an eternal punishment caused by an eagle eating his liver, which would replenish itself every day. Indeed, after partial hepatectomy, which leads to removal of up to 70% of the liver, liver mass and functions become significantly restored within a few days. Mechanisms underlying restoration of liver mass and function in response to hepatectomy have been elegantly reviewed elsewhere (Michalopoulos and DeFrances, 1997; Michalopoulos, 2007). In this review, I focus on adult liver regeneration in response to tissue damage that can normally challenge the liver, in line with its central role in nutrient metabolism and detoxification in the adult body.

The adult liver is a slowly self-renewing organ and does not exhibit a defined stem-cell compartment that maintains homeostasis, opposite to other organs that have excellent regenerative capacity, including intestine, stomach, and skin. A great number of experimental models indicate that cellular plasticity, intended as the capacity of liver cells to adopt alternative fates and functions in response to environmental changes, determines efficient adult liver regeneration following damage (Tetteh et al., 2015; Aloia et al., 2016; Kopp et al., 2016; Gadd et al., 2020). A key question is: what molecular mechanisms regulate cellular plasticity and cell-fate changes to achieve efficient liver regeneration upon injury? In 1957, Conrad Hal Waddington postulated that the epigenetic landscape contributes to determine cell-fate decisions and acquisition of cell-identity during embryo development (Waddington, 1957). Increasing evidence indicates that the epigenetic landscape is dynamically regulated in the adulthood and allows cell-fate changes and cellular plasticity in adult tissues in response to environmental cues and tissue injury (Takahashi and Yamanaka, 2015; Rajagopal and Stanger, 2016; Flavahan et al., 2017; Paksa and Rajagopal, 2017). Of note, here I refer to epigenetic mechanisms as modifications of the chromatin (the ensemble of DNA and histones), which are involved in the regulation of gene transcription. Importantly, epigenetic mechanisms are inheritable by the cell progeny and reversible in response to environmental changes. One intriguing hypothesis is that dynamic regulation of epigenetic mechanisms might represent an efficient way for liver cells to respond to injury, repair the damaged tissue, and return to the homeostatic state once the injury is resolved. Supporting this hypothesis, our data indicated that transient epigenetic remodelling, mediated by the methylcytosine dioxygenase TET1, triggers liver cellular plasticity and contributes to liver regeneration following injury (Aloia et al., 2019). Here, I review the role of several chromatin modifiers in the regulation of cell-fate changes required for liver epithelial regeneration after injury in mouse models (Table 1). In addition, I address the interplay between epigenetics and metabolism in liver regeneration and chronic liver disease and discuss how epigenetic alterations may contribute to liver disease and cancer.

**TABLE 1:** Role of selected chromatin modifiers in liver epithelial regeneration following tissue injury.

**Name**	**Function**	**Liver cell type**	**Role in liver epithelial regeneration upon injury**
ARID1A *AT-Rich Interaction Domain 1A*	DNA interacting subunit of the chromatin remodelling complex SWI/SNF	Hepatocyte	• Blocks proliferation and stabilises the differentiated state (Sun et al., 2016). • Facilitates YAP-dependent transcriptional activity and induction of bipotent liver progenitors (Li et al., 2019).
DNMT1 *DNA (cytosine-5)-methyltransferase 1*	DNA methyltransferase (maintenance of 5-methycytosine during replication)	Hepatocyte	• Protects from DNA damage and senescence. Deletion causes differentiation defects (Kaji et al., 2016).
EZH 1/2 *Enhancer of zeste homolog 1/2*	Enzymatic subunits of the Polycomb Repressive Complex 2, PRC2	Hepatocyte	• Repress cell-cycle inhibitors (Bae et al., 2015).
HDAC1 *Histone* deacetylase 1	Histone deacetylase	Cholangiocyte	• Promotes differentiation of bipotent liver progenitors into hepatocytes, repressing the expression of Sox9 (Ko et al., 2019).
TET1 *Ten-eleven translocation methylcytosine dioxygenase 1*	Oxidation of 5-methylcytosine (5mC) to 5-hydroxymethylcytosine (5hmC), 5-formylcytosine (5fC) and 5-carboxylcytosine (5caC)	Cholangiocyte	• Enables de-differentiation into bipotent liver progenitors, promoting the expression of stem-cell and regenerative genes, including members and targets of the YAP signalling pathway (Aloia et al., 2019).
UHRF1 *Ubiquitin Like With PHD And Ring Finger Domains 1*	Recruitment of DNMT1 to chromatin; Binding to H3K9me3	Hepatocyte	• Depletion results in epigenetic compensation that facilitates the induced expression of cell-cycle and regenerative genes (Wang et al., 2019).

## Hepatocytes and Cholangiocytes Exhibit Remarkable Plasticity in Response to Liver Injury

Two epithelial cell types are present in the adult liver: (i) hepatocytes, which represent >60% of total liver cells and perform most of the metabolic and detoxification functions of the organ and (ii) cholangiocytes, which represent 3–5% of total liver cells and form biliary ducts that collect the bile produced by the hepatocytes and export it to the intestine for digestive purposes (Ober and Lemaigre, 2018). Both hepatocytes and cholangiocytes exhibit remarkable plasticity in response to injury. The role of epithelial plasticity in liver regeneration has been elegantly reviewed by Stuart Forbes and colleagues (Gadd et al., 2020).

Increasing evidence indicates that the vast majority of the hepatocytes, if not all, can acquire proliferation to ensure efficient restoration of liver mass and functions (Chen et al., 2020; Matsumoto et al., 2020; Monga, 2020; Sun et al., 2020). Upon liver injury induced by chemicals or toxins, hepatocytes can trans-differentiate into cholangiocytes (Yanger et al., 2013) or de-differentiate into bipotent liver progenitors, capable of generating both liver epithelial cell types (Tarlow et al., 2014; Yimlamai et al., 2014; Han et al., 2019). Liver injury can also trigger cholangiocyte proliferation and expansion in the liver parenchyma, a process known as ductular reaction (Roskams et al., 2004). Importantly, cholangiocytes can de-differentiate *in vivo* into bipotent liver progenitors that give rise to both hepatocytes and cholangiocytes (Itoh and Miyajima, 2014; Ko et al., 2020; So et al., 2020). The role of cholangiocytes in liver regeneration has been extensively debated. Several experimental models indicated that hepatocytes are solely responsible for restoration of the epithelial compartment after liver injury and the contribution of cholangiocytes is neglectable (Malato et al., 2011; Schaub et al., 2014; Yanger et al., 2014; Font-Burgada et al., 2015; Lin et al., 2018). However, different mouse models demonstrated that cholangiocytes significantly contribute to liver regeneration when hepatocytes are compromised. Data from Lu et al. (2015) indicated that cholangiocytes have liver repopulation capacity upon transplantation into adult mouse liver that shows extensive hepatocyte senescence and necrosis induced by genetic deletion of the E3 ubiquitin ligase *Mdm2*. Moreover, lineage-tracing experiments demonstrated that cholangiocytes can restore from 10 to 70% of total liver hepatocytes following diet-induced liver injury in mouse models exhibiting genetic deletion of *β1-integrin* (Raven et al., 2017) or *β-catenin* (Russell et al., 2018) or over-expression of p21 (Raven et al., 2017) in hepatocytes or after prolonged liver injury in the absence of genetic alterations that might compromise hepatocyte functions (Deng et al., 2018; Manco et al., 2019).

The capacity of both hepatocytes and cholangiocytes to acquire a bipotent progenitor state relies on their origin from a common embryonic progenitor, the hepatoblast (Zaret and Grompe, 2008; Ober and Lemaigre, 2018). Of note, chromatin modifications such as trimethylation of lysine 9 and 27 of histone H3 (H3K9me3 and H3K27me3) play a crucial role in the establishment of hepatoblast identity (Nicetto et al., 2019), supporting the relevance of epigenetic mechanisms in liver cell-identity and cell-fate decisions. In the following sections, I examine the role of several epigenetic modifiers in liver regeneration mediated by adult hepatocytes and cholangiocytes following tissue damage.

## Epigenetic Mechanisms Regulating Hepatocyte-Mediated Liver Regeneration

The hepatocytes play a crucial role in adult liver regeneration after injury (Malato et al., 2011; Schaub et al., 2014; Yanger et al., 2014; Font-Burgada et al., 2015; Lin et al., 2018). Arid1a, a DNA interacting subunit of the SWI/SNF chromatin remodelling complex, has been shown to regulate hepatocyte plasticity in mouse models carrying liver-specific deletion of *Arid1a* (by using an *Albumin*-Cre/*Arid1a* flx/flx mouse line) or conditional deletion of *Arid1a* in hepatocytes (mediated by infection with an AAV-Cre in *Arid1a* flx/flx mice) (Sun et al., 2016; Li et al., 2019). Sun et al. (2016) found that *Arid1a* deletion increased hepatocyte proliferation and reduced necrosis, inflammation, and fibrosis in response to liver damage induced by carbon tetrachloride, CCl4. Moreover, *Arid1a* deletion resulted in accelerated weight recovery and extended survival following treatment with the toxic diet 3,5-diethoxycarbonyl-1,4-dihydrocollidine, DDC (6 weeks). Depletion of Arid1a following damage, in the recovering liver after a 12-week treatment with CCl4 or a 4-week treatment with DDC, reduced fibrosis and accelerated regain of normal weight. Thus, these data indicated that Arid1a deletion facilitated liver recovery from damage. At the molecular level, Sun et al. (2016) showed that Arid1a genomic binding might facilitate chromatin accessibility to key transcription factors that repress proliferation (e.g., E2F4) and maintain the hepatocyte differentiated cell state, such as C/EBPa and Hnf4a (Sun et al., 2016). Together, these findings suggested that Arid1a might impair liver regeneration by stabilising the hepatocyte differentiated state and reducing hepatocyte plasticity (Sun et al., 2016). This hypothesis is challenged by the findings of Li et al. (2019), who showed that conditional *Arid1a* deletion resulted in a significant reduction of Hnf4a^+^/Sox9^+^ bipotent liver progenitors in response to DDC treatment (2 weeks). At the molecular level, they found that Arid1a determined increased chromatin accessibility in the homeostatic liver, which facilitates YAP genomic binding after liver injury. YAP transcriptional activity was previously shown to promote hepatocyte de-differentiation into liver progenitors (Yimlamai et al., 2014). Consistently, Li et al. (2019) found that the number of YAP^+^/Sox9^+^ liver progenitors was significantly reduced in *Arid1a*-depleted mice upon over-expression of a constitutively active YAPS127A. Of note, they found that *Arid1a* deletion resulted in hepatomegaly, hepatocyte hypertrophy, and impaired liver functions upon DDC-mediated liver damage, whereas its depletion following DDC-induced damage did not affect liver recovery (Li et al., 2019). The different role of Arid1a in liver regeneration and recovery after injury reported by Sun et al. (2016) and Li et al. (2019) could be explained by the different length of DDC treatment and recovery, which might result in different levels of tissue injury at the time of the analyses performed (4–6 weeks DCC followed by 0–10 days recovery vs 2 weeks DDC followed by 8 weeks recovery, respectively). The different length of injury/recovery might result in a different timing of Arid1a binding to DNA and SWI/SNF-mediated chromatin remodelling activity. This, in turn, might influence the induction of hepatocyte de-differentiation into progenitors and their return to the homeostatic state. Of note, in their experimental conditions, Sun et al. (2016) reported increased hepatocyte proliferation following *Arid1a* depletion, whereas Li et al. (2019) did not observe a significant difference. This has important consequences on hepatocyte plasticity, since increased proliferation of mature hepatocytes is likely to determine faster regeneration, thus reducing the requirement of hepatocyte de-differentiation into progenitors.

To achieve efficient liver regeneration, it is crucial that adult hepatocytes maintain their functions during proliferation and cell division. Bae et al. (2015) found that EZH1 and EZH2 regulated hepatocyte proliferation and promoted increased mouse survival following CCl4. EZH1/2 are the catalytic subunits of the Polycomb repressive complex 2 (PRC2), which result in trimethylation of lysine 27 of histone 3 (H3K27me3), an epigenetic mark mainly associated to gene repression. Bae et al. (2015) found that key target genes of EZH1/2 are the cell-cycle inhibitors *Cdkn2a* and *Cdkn2b*, which became up-regulated in *Ezh1/2* knock-out (KO) livers. Consistent with the importance of hepatocyte proliferation and division in liver regeneration, epigenetic regulation mediated by the maintenance DNA methyltransferase DNMT1 and its regulator UHRF1 has been shown to play an important role in hepatocyte viability and regenerative capacity (Kaji et al., 2016; Wang et al., 2019). Kaji et al. (2016) and Wang et al. (2019) performed liver-specific deletion of *Dnmt1* and *Uhrf1*, respectively, crossing *Albumin*-Cre with specific flx/flx mouse lines. Wang et al. (2019) showed that *Uhrf1* deletion resulted in global hypomethylation, which induced redistribution of repressive H3K27me3 from promoters to transposable elements to block their aberrant expression. This has implications in liver regeneration, since reduced H3K27me3 levels at promoter facilitated the induced expression of cell-cyle and regenerative genes (Wang et al., 2019). Kaji et al. (2016) showed that *Dnmt1* deletion induced liver fibrosis and inflammation and resulted in hepatocyte senescence. Intriguingly, *Dnmt1* deletion resulted in maturation defects and lack of expression of the cytochrome *Cyp2e1*, which is responsible for the bioactivation of CCl4, thus making *Dnmt1* KO mice resistant to liver injury induced by CCl4 (Kaji et al., 2016). Of note, Bae et al. (2015) and Kaji et al. (2016) showed that impaired hepatocyte regenerative capacity or tissue damage caused by hepatocyte senescence and necrosis triggered ductular reaction.

These findings highlight the relevance of epigenetic mechanisms in the regulation of hepatocyte viability, proliferation, and plasticity. Further work will determine how dynamic epigenetic regulation influences the balance between proliferation of mature hepatocytes and hepatocyte de-differentiation into progenitors and how those two processes act to coordinate liver response to injury to achieve efficient regeneration.

## Epigenetic Mechanisms Regulating Cholangiocyte-Mediated Liver Regeneration

Cholangiocyte expansion and de-differentiation into liver progenitors have been extensively reported in a variety of injury models in mammals (Okabe et al., 2009; Sackett et al., 2009; Dorrell et al., 2011; Shin et al., 2011; Espanol-Suner et al., 2012; Huch et al., 2013). Lineage-tracing experiments in mouse models demonstrated a crucial role for cholangiocytes in adult liver regeneration when hepatocyte response to injury is compromised by genetic alterations or following severe and prolonged liver damage (Raven et al., 2017; Deng et al., 2018; Russell et al., 2018; Manco et al., 2019).

What mechanisms regulate cholangiocyte plasticity? Our data demonstrated that upon liver injury, cholangiocytes undergo epigenetic remodelling mediated by the methylcytosine dioxygenase TET1 (Aloia et al., 2019). TET1/2/3 oxidise the repressive DNA mark 5-methylcytosine into 5-hydroxymethylcytosine (5hmC). 5hmC can act as a stable epigenetic mark associated with gene activation or represent an intermediate of complete de-methylation after further oxidation catalysed by TET proteins (Rasmussen and Helin, 2016). We found that TET1 was lowly expressed in homeostatic adult cholangiocytes and was up-regulated following DDC-mediated liver injury *in vivo* vs healthy liver, opposite to *Tet2/3*, which became down-regulated. By using a *Tet1* hypomorphic mouse, we found that TET1 reduced levels impaired cholangiocyte proliferation after acute DDC treatment (5 days) and resulted in liver fibrosis after prolonged DDC-mediated liver injury (∼8 weeks including off-treatment intervals) (Aloia et al., 2019). Lineage-tracing experiments demonstrated that TET1 was required for the formation of cholangiocyte-derived hepatocyte regenerative clusters following DDC-mediated liver injury (Aloia et al., 2019), using a liver injury model developed by Raven et al. (2017). Together, our findings indicated that TET1 triggers cholangiocyte plasticity in response to liver injury and is required for efficient liver epithelial regeneration. At the molecular level, we found that TET1 regulates the expression of stem-cell genes that identify hepatoblasts (e.g., *Lgr5*; Prior et al., 2019) and adult human and mouse bipotent liver progenitors (e.g., *Trop2*; Okabe et al., 2009; Aizarani et al., 2019). Consistently, TET1 was required for the establishment of mouse intrahepatic cholangiocyte organoids, which exhibit induced expression of stem-cell genes (e.g., *Lgr5* and *Trop2*) and can differentiate into hepatocytes (Huch et al., 2013). Moreover, genome-wide TET1 DamID-sequencing (van den Ameele et al., 2019) in intrahepatic cholangiocyte organoids revealed that TET1 regulates the expression of components and targets of signalling pathways required for liver regeneration, including YAP (Yimlamai et al., 2014; Pepe-Mooney et al., 2019; Planas-Paz et al., 2019). Together, our data suggested that TET1 enables de-differentiation of cholangiocytes into bipotent liver progenitors both *in vitro and in vivo* to ensure efficient liver regeneration and organoid formation (Aloia et al., 2019).

Of note, the increase in *Tet1* levels after liver injury *in vivo* (day 3 following DDC treatment) was only transient, since *Tet1* expression decreased at the peak of DDC-mediated liver damage (day 5). In line with this, we found that 5hmC levels were transiently increased in cholangiocytes *in vivo* at day 3 at the transcriptional start site of >3000 genes, which are mainly involved in biological processes such as development, chromatin modifications, and cell-cycle (Aloia et al., 2019). Therefore, this suggested that dynamic epigenetic remodelling *in vivo* allows cholangiocytes to both respond to liver injury and return to the homeostatic state to avoid the detrimental effects of an aberrant progenitor response. Supporting this hypothesis, Lgr5^+^ cells have been identified as tumour initiating cells in mouse liver cancer (Cao et al., 2020) and YAP can induce liver cancer (Dong et al., 2007).

What epigenetic mechanisms regulate cholangiocyte differentiation into hepatocytes? Following experiments in zebrafish that led to the identification of a regenerative role for Hdac1 by repressing the expression of Sox9 and modulating the Notch signalling, Ko et al. (2019) showed that the HDAC inhibitor MS-275 impaired cholangiocyte-mediated hepatocyte regeneration in a mouse model developed by Russell et al. (2018). Of note, MS-275 treatment did not affect cholangiocyte proliferation and ductular reaction (Ko et al., 2019), thus suggesting that Hdac1 specifically regulates cholangiocyte differentiation into hepatocytes without affecting cholangiocyte response to liver injury.

These findings highlight the relevance of dynamic epigenetic regulation of cholangiocyte plasticity for liver regeneration in response to severe or persistent liver injury that compromises the hepatocytes. Of note, ductular reaction is often observed in human chronic liver disease, concomitantly with massive hepatocyte alterations and necrosis (Ko et al., 2020). Therefore, understanding the epigenetic mechanisms that regulate cholangiocyte plasticity might indicate novel therapeutic strategies aimed at stimulating cholangiocyte regenerative capacity to ameliorate human chronic liver disease.

## The Interplay Between Epigenetics and Metabolism in Liver Regeneration and Disease

Epigenetic mechanisms are often regulated by metabolic inputs (Etchegaray and Mostoslavsky, 2016). For example, TET1 epigenetic activity is dependent on 2-oxoglutarate, which is an intermediate of the Krebs cycle (Rasmussen and Helin, 2016). The adult liver is the central organ of the body for nutrient metabolism and presents metabolic zonation with specialised metabolic functions. Liver zonation follows decreasing oxygen and nutrient gradient across the liver lobule from the portal triad (formed by the hepatic artery, portal vein, and biliary ducts), to the central vein, where the blood meets the systemic circulation (Kietzmann, 2017). This suggests that different types and durations of liver injuries may affect different metabolic zones and trigger specific epigenetic mechanisms, which, in turn, promote distinct cellular response to damage. Importantly, metabolic alterations might cause chronic liver disease. For example, metabolic disorders are associated with the most common form of human chronic liver disease, non-alcoholic fatty liver disease (NAFLD). Patients with NAFLD exhibit increased fat accumulation and can present significant inflammation, named as non-alcoholic steatohepatitis (NASH) (Benedict and Zhang, 2017). At late stages, chronic liver disease is characterised by cirrhosis, which accounts for >1 million deaths per year worldwide and can predispose to liver cancer (Asrani et al., 2019). Therefore, it is reasonable to speculate that metabolic dysfunctions induce epigenetic alterations that, in turn, might impair liver regeneration capacity and exacerbate liver disease and favour its progression towards liver cirrhosis. The interplay between epigenetics and metabolism is bidirectional, since altered epigenetic mechanisms may also cause metabolic dysfunctions and trigger liver disease. A circadian rhythm was shown to determine chromatin recruitment of the histone deacetylase Hdac3, which in turn controls the expression of genes related to lipid metabolism, and determines the balance between gluconeogenesis and lipid synthesis (Feng et al., 2011; Sun et al., 2012). Consistently, *Hdac3* deletion in mouse resulted in hepatic steatosis (Feng et al., 2011). In addition, Arid1a was found to regulate lipogenesis and fatty acid oxidation and its deletion was implicated in NASH in mouse (Fang et al., 2015; Moore et al., 2019).

Together, this suggests that investigating the complex interplay between epigenetics and metabolism will represent an important step to understand how the liver achieves efficient regeneration and identify the molecular mechanisms that influence initiation and progression of chronic liver disease.

## Discussion

**FIGURE 1:**
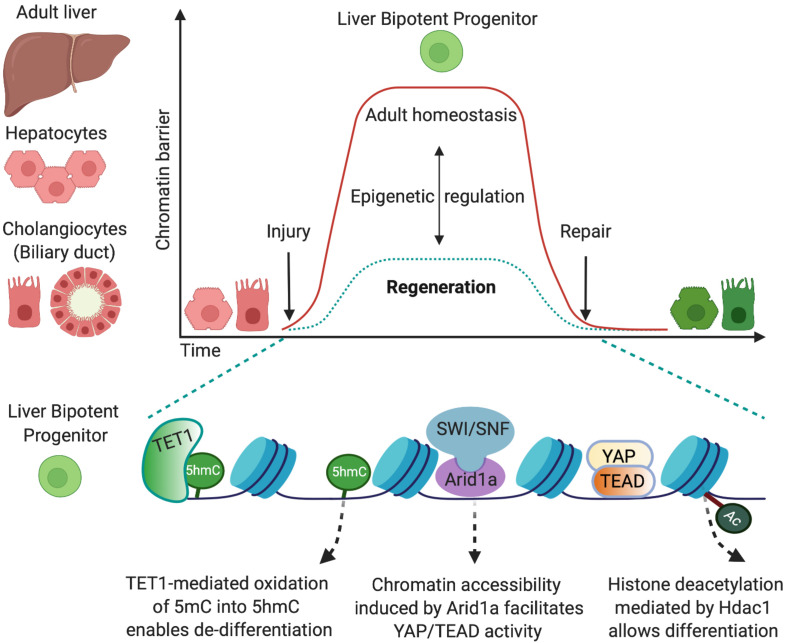
Epigenetic mechanisms that allow cell-fate changes into bipotent liver progenitors. The adult liver is formed by two epithelial cell types, hepatocytes and cholangiocytes, which derive from a common bipotent embryonic progenitor, the hepatoblast. In the adulthood, in homeostatic conditions, the epigenetic landscape preserves cell-identity by maintaining chromatin conformations that stabilise the differentiated state (red). Upon injury, both adult hepatocytes and cholangiocytes can de-differentiate into bipotent liver progenitors that give rise to both liver epithelial cell types (green) and restore the liver epithelial compartment. This cell-fate change into progenitors is enabled by epigenetic mechanisms that determine permissive chromatin states, including increased chromatin accessibility mediated by Arid1a and the chromatin remodelling complex SWI/SNF (Li et al., 2019) and oxidation of 5-methycytosine (5mC) into 5-hydromethylcytosyne (5hmC) mediated by the methylcytosine dioxygenase TET1 (Aloia et al., 2019). Such permissive states facilitate the chromatin binding of the transcriptional machinery (e.g., YAP/TEAD) that promotes the establishment of liver progenitor identity. Importantly, dynamic epigenetic regulation allows liver epithelial cells to return to the homeostatic state once the injury is resolved and the tissue is repaired. In this regard, TET1 and 5hmC levels are only transiently enriched in cholangiocytes at early stages upon liver injury (Aloia et al., 2019), and histone deacetylation mediated by Hdac1 is required for differentiation of cholangiocyte progenitors into hepatocytes (Ko et al., 2019).

In this review, I have examined the role of several chromatin modifiers that regulate epithelial cell-fate changes to achieve efficient liver regeneration after injury in mouse models (Figure 1). It is worth mentioning that whether mouse models allow recapitulating several aspects of human liver regeneration and disease, species-specific difference should be taken in account, and the epigenetic mechanisms identified in mouse models should be validated also in human models such as cell lines, organoid systems, or primary specimens.

The expression levels of chromatin modifiers and genomic distribution of their epigenetic marks are often altered in human chronic liver disease and liver cancer (Hardy and Mann, 2016). For example, loss of function mutations in *ARID1A* have been reported in >16% of human hepatocellular carcinoma (Guichard et al., 2012). Arid1a was shown to have a dual role as both oncogene and tumour suppressor in mouse, supporting the hypothesis that epigenetic mechanisms determine specific cellular phenotypes according to the context of the surrounding tissue. Specifically, Arid1a triggered liver cancer initiation by increasing reactive oxygen species, whereas its loss in pre-existing tumours promoted cancer growth and metastasis (Sun et al., 2017). UHRF1 is often over-expressed in human liver cancer and high UHRF1 levels are associated with a poor prognosis. Interestingly, UHRF1 over-expression was shown to induce hypomethylation in human liver cancer (Mudbhary et al., 2014), as observed upon its depletion in mouse, where it induced epigenetic compensation (Wang et al., 2019). Further studies will determine the relevance of dynamic epigenetic remodelling in human liver cancer.

Pharmacological interventions based on drugs targeting epigenetic activity have a huge potential for the development of therapeutic strategies in human disease and cancer (Cheng et al., 2019). Therefore, advances in our knowledge of the epigenetic mechanisms that regulate liver plasticity might open new avenues for the treatment of chronic liver disease and liver cancer. In this regard, 3D liver organoid cultures derived from adult liver biopsies or induced pluripotent stem cells have proven reliable models of homeostatic and regenerative cholangiocytes (Huch et al., 2013, 2015; Sampaziotis et al., 2017; Aloia et al., 2019; Rimland et al., 2020) and hepatocytes (Hu et al., 2018; Peng et al., 2018), steatohepatitis (Ouchi et al., 2019; Ramli et al., 2020), and primary liver cancer (Broutier et al., 2017; Nuciforo et al., 2018), thus representing promising platforms to uncover the molecular mechanisms underlying liver regeneration, disease, and cancer.

## Author Contributions

The author confirms being the sole contributor of this work and has approved it for publication.

## Conflict of Interest

The author declares that the research was conducted in the absence of any commercial or financial relationships that could be construed as a potential conflict of interest.
